# Profiles of institutional departments affect operative outcomes of eight gastroenterological procedures

**DOI:** 10.1002/ags3.12431

**Published:** 2021-02-20

**Authors:** Hiroyuki Konno, Kinji Kamiya, Arata Takahashi, Hiraku Kumamaru, Yoshihiro Kakeji, Shigeru Marubashi, Kenichi Hakamada, Hiroaki Miyata, Yasuyuki Seto

**Affiliations:** ^1^ The Japanese Society of Gastroenterological Surgery Database Committee Tokyo Japan; ^2^ Hamamatsu University School of Medicine Hamamatsu Japan; ^3^ Second Department of Surgery Hamamatsu University School of Medicine Hamamatsu Japan; ^4^ Department of Health Policy and Management School of Medicine Keio University Tokyo Japan; ^5^ Department of Healthcare Quality Assessment Graduate School of Medicine The University of Tokyo Tokyo Japan; ^6^ The Japanese Society of Gastroenterological Surgery Tokyo Japan

**Keywords:** National clinical database, observed to expected (O/E) operative mortality ratio, operative mortality, profiles of institutional departments, questionnaire

## Abstract

**Aim:**

We evaluated the association of profiles of institutional departments with operative outcomes of eight major gastroenterological procedures.

**Methods:**

We administered a 15‐item online survey to 2634 institutional departments in 2016 to investigate the association of questionnaire responses with operative mortality for the procedures. The proportions of conditions met were listed according to institutional volume and classified according to annual operative cases in 1464 departments. Group A included departments with annual performance of <40 cases of the eight procedures, B 40‐79 cases, C 80‐199 cases, D 200‐499 cases, and E ≥ 500 cases. We evaluated the number of conditions met for 10 of 15 items that could be improved by efforts of institutional departments, to assess whether the profiles of institutional departments had impacts on operative mortality. We built a multivariable logistic regression model for operative mortality with facilities categorized based on the number of conditions met and procedure‐specific predicted mortality as explanatory variables using generalized estimating equation to account for facility‐level clustering. We also examined how operative outcomes differed between facilities meeting nine or more conditions and those that did not.

**Results:**

We recognized meeting nine out of the 10 conditions as being a good indicator for having appropriate structural and process measures for gastroenterological surgery. The facilities meeting nine or more of the conditions had better operative mortality for all eight procedures.

**Conclusions:**

Our findings reveal that the profiles of institutional departments can reflect the outcomes of gastroenterological surgery in Japan.

## Graphical abstract

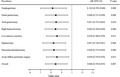

## 1 INTRODUCTION

In a previous study,^1^ we reported that board‐certified surgeons of gastroenterological surgery (BCS‐Gs) contribute to favorable outcomes of gastroenterological surgery in Japan based on analysis of the National Clinical Database (NCD), a nationwide web‐based data entry system for eight procedures of gastroenterological surgery, consisting of esophagectomy (Eso), distal gastrectomy (DG), total gastrectomy (TG), right hemicolectomy (RHC), low anterior resection (LAR), hepatectomy (Hx), pancreaticoduodenectomy (PD), and acute diffuse peritonitis surgery (ADP). In our previous study, the number of BCS‐Gs in an institute was shown to have a significant correlation with operative mortality. To be specific, the ratio of observed to expected (O/E) operative mortality in institutions with four or more BCS‐Gs was less than 1.0 for all procedures. Multivariable logistic regression showed that the number of institutional BCS‐Gs was a predictor of operative mortality. As a result, we revealed that the number of institutional BCS‐Gs is a surrogate marker of operative mortality.

In the present study, we investigated the association of profiles of institutional departments assessed by an online questionnaire survey with operative outcomes. The NCD, in which BCS‐Gs are required to register their cases, commenced patient registration in January 2011. The gastroenterological surgery section of the NCD requires detailed input items for the eight major procedures. Using NCD data from 2011 regarding nationwide outcomes for the eight procedures, risk models of operative mortality^2^, ^3^, ^4^, ^5^, ^6^, ^7^, ^8^, ^9^ and morbidity^10^, ^11^, ^12^, ^13^, ^14^, ^15^, ^16^, ^17^ have been developed. These surgical risk models likely represent the current nationwide status in Japan because they are free of the patient selection bias that can occur in randomized controlled trials. The mortalities for all eight procedures seem acceptable as nationwide outcomes, being satisfactorily low compared with those reported in other countries.^18^, ^19^, ^20^

Although requirements for application to become a board‐certified institute are authorized by the Japanese Society of Gastroenterological Surgery (JSGS), it has not been reported whether the profiles of institutional departments influence operative outcomes. In the present study, we investigated the impact of profiles of institutional departments on operative outcomes in Japan.

## 2 MATERIALS AND METHODS

### 2.1 Board certification system of the JSGS

The board certification system of the JSGS consists of board‐certified training institutions and BCS‐Gs. Table 1 shows the requirements for JSGS‐certified institutions. In Japan, there are approximately 2000 JSGS‐certified institutions. Among 10 mandatory factors, having performed 120 essential major surgeries in the past 3 years is required of applicants for board certification.

**TABLE 1:** Requirements of application for a board‐certified training institute authorized by the JSGS

1	Have performed 600 or more the gastroenterological surgeries determined by the Certified Committee (more than 120 of them essential major surgery^a^) in the last three years.
2	Have a JSGS‐certified two supervisory surgeons, or a BCS‐G other than one supervisory surgeon.
3	Be capable of training for overall gastroenterological surgery.
4	Have a well‐facilitated medical recording system in the institute.
5	Have an established ethical committee or be able to refer to other organizations when any ethics‐related issues arise.
6	Have organized gastroenterology‐related educational events (such as case conferences and mortality conferences) on a regular basis.
7	Have published more than three studies in any scientific journal or annual congress in the last three years.
8	Be capable of accepting physicians who wish to become a BCS‐G.
9	Accept attendance at annual congresses or educational seminars as a part of training.
10	Be capable of rigorous investigation of the medical experience of applicants for the BCS‐G.

Abbreviations: BCS‐Gs, board‐certified surgeons of gastroenterological surgery; JSGS, Japanese Society of Gastroenterological Surgery.

^a^ Surgery for esophageal cancer, distal gastrectomy, total gastrectomy, surgery for colon cancer, surgery for rectal cancer, surgery for bowel obstruction, partial hepatectomy, two or more segmentectomies of the liver, pancreaticoduodenectomy.

### 2.2 Data source and registry platform

The NCD was implemented in 2010 by 10 surgical societies including the Japan Surgical Society and the JSGS. Registration to the NCD through online data collection system was initiated in 2011. This large nationwide database covers more than 95% of surgeries throughout Japan and more than 11 300 000 cumulative cases were registered by the end of 2018^21^. We used data from the NCD gastroenterological division for the present study.

### 2.3 Questionnaire survey

We conducted an online questionnaire survey from February to March 2016 using the NCD system targeting all 2634 institutional departments that performed at least one gastroenterological surgery in 2015. The 15 items in the questionnaire are shown in Table 2. The questions were created to capture the departments’ structural as well as procedural characteristics on how they care for their surgical patients.

**TABLE 2:** Questionnaire items

Q1	Do you decide the adaptation of elective surgery by preoperative conference?
Q2	Is Cancer Board held in your institution?
Q3	Is MM conference held in your institution?
Q4	Do you utilize the NCD feedback system for clinical treatment?
Q5	Is team treatment system built in your institution?
Q6	Is your institution education institutional (a university institutional, a clinical training institutional, a research institutional, etc)?
Q7	Is there ICU in your institution?
Q8	Is there ICT in your institution?
Q9	Is there NST in your institution?
Q10	Is there medical safety committee in your institution?
Q11	Are you checking (not less than 90% of enforcement rate) the WHO safe check list when starting a surgery?
Q12	How many BCS‐Gs are there in your institution?
Q13	How many numbers of surgery is performed per year in your institution?
Q14	How many certified nurses are there in your institution?
Q15	How many institutional beds are there in your institution?

Abbreviations: BCS‐Gs, board‐certified surgeons of gastroenterological surgery; ICT, infection control team; ICU, intensive care unit; MM, mortality and morbidity; NCD, National Clinical Database; NST, nutrition support team; WHO, World Health Organization.

### 2.4 Patients

We selected all patients who had undergone any of the eight procedures between 1 January and 31 December 2015 from the NCD registry. We identified 137 349 patients comprising 6060 Eso, 37 821 DG, 18 696 TG, 22 853 RHC, 22 498 LAR, 7439 Hx, 10 578 PD, and 13 037 ADP (including duplicates). The main outcomes of interest in the study were operative death, defined as death within the index hospitalization period regardless of length of hospital stay (up to 90 days), as well as any death within 30 days post surgery.

### 2.5 Classification of institutional departments based on annual case numbers

The strong associations between volume and operative outcomes have been reported elsewhere.^22^, ^23^, ^24^, ^25^ In the present study, the relationships between departmental annual case numbers of the eight procedures and departmental factors were investigated, to confirm the impact of the number of annual cases on operative outcomes. In accordance with the requirements for board certification (Table 1), institutional departments were classified into five categories based on annual operative case numbers in their institutes. Group A was defined as institutional departments with annual performance of <40 cases of the eight procedures, Group B 40‐79 cases, Group C 80‐199 cases, Group D 200‐499 cases, and Group E ≥500 cases.

### 2.6 Ten improvable departmental characteristics

Ten of the 15 items were selected to investigate the relationship between proportion of affirmative responses and operative outcomes. We chose these 10 items because they showed a positive association with operative outcomes and are conditions that can be improved by efforts of institutional departments or their institutes. The 10 items were: holding a preoperative conference, having a cancer board, holding a mortality and morbidity (MM) conference, utilizing the NCD feedback system^26^ for departmental performances, using the World Health Organization (WHO) safety checklist, establishing a team treatment system, having an infection control team (ICT), having a nutrition support team (NST), having BCS‐Gs on site, and having certified nurses (Table 3).

**TABLE 3:** Ten evaluation items

Evaluation item
The adaptation of elective surgery is decided by preoperative conference.
Cancer Board is held.
MM conference is held.
NCD feedback system is used for clinical treatment.
Team treatment system is built.
ICT is installed.
NST is installed.
WHO safe check list is checked when starting a surgery.
There are two or more BCS‐Gs.
There is certified nurse.

Abbreviations: BCS‐Gs, board‐certified surgeons of gastroenterological surgery; ICT, infection control team; MM, mortality and morbidity; NCD, National Clinical Database; NST, nutrition support team; WHO, World Health Organization.

### 2.7 Statistical analysis

For each question in the questionnaire, we assessed the number and percentage of departments by their responses. We also evaluated the crude operative mortality and O/E ratio for the procedures performed at the departments by their responses. The O/E ratios were calculated by dividing the observed operative mortality by the predicted operative mortality using eight risk models previously created and reported using NCD gastroenterological data.^2^, ^3^, ^4^, ^5^, ^6^, ^7^, ^8^, ^9^, ^11^ For cases that underwent more than two of the eight procedures, the risk model for the more invasive procedure was used for the estimation.

We assessed the responses to the questionnaire by the facility volume group as described above. To assess the association between number of conditions which the departments met among the 10 improvable items and operative mortality, we built a multivariable logistic regression model for operative mortality with facilities categorized based on the number of conditions met (0‐1 being the lowest and 10 being the highest) and procedure‐specific predicted mortality as explanatory variables using a generalized estimating equation to account for facility‐level clustering. Furthermore, we also assessed the association of meeting nine or more of the conditions and operative outcomes by multivariable logistic regression analysis with each patient’s baseline predicted risk as well as hospital case volume. This analysis was conducted among all patients, as well as by surgical procedures. We have presented the baseline variables used to estimate these predicted mortalities for each procedure, as in Appendix S1.

All tests were two‐sided, and values of *P* < .05 were considered statistically significant. All analyses were performed using IBM SPSS version 24 (IBM Corp., Armonk, NY, USA).

## 3 RESULTS

Among 2634 institutional departments, 1579 responded to the questionnaire (59.9%). Among these, we selected 1464 institutional departments with at least one registered patient who underwent one of the eight selected procedures in 2015. As a result, 113 453 cases were included in the analysis. Table 4 shows the responses to the 15 questions among the 1464 institutional departments. Preoperative conference, cancer board, and MM conference were present in 65.1%, 61.5%, and 50.9% of the institutional departments, respectively. An ICT, NST, and medical safety committee were established in over 90% of the institutes, and the WHO checklist was completed in 83.1% of institutional departments when starting a surgery. Departments that held a preoperative conference, had a cancer board, had a team treatment system, used the NCD feedback system, had an ICT, or had an NST exhibited better observed mortality than departments without these characteristics, while existence of a medial safety committee was not associated with reduced mortality. In total, 68.3% of departments had two or more BCS‐Gs, and the O/E ratios in these departments were significantly lower than those in departments without a BCS‐G or with only one BCS‐G. Furthermore, 10.2% of institutional departments without a certified nurse in their institution had the worst O/E ratio (1.38) among the four groups.

**TABLE 4:** Response distribution for each QI and relationship of each QI result with operative mortality, risk‐adjusted operative mortality, and mortality O/E ratio

Questionnaire item	Institutional departments		Crude operative mortality (%)	*P* Value	Predicted operative mortality (%)	Mortality O/E ratio	95%CI	
	N	%					lower	upper
Q1								
Preoperative conference								
Yes	953	65.1	2.4	<.001	3.0	0.81	0.78	0.84
No	511	34.9	2.9		2.7	1.05	0.97	1.14
Q2								
Cancer Board								
Yes	900	61.5	2.4	<.001	3.0	0.81	0.78	0.85
No	564	38.5	3.1		2.9	1.08	0.99	1.17
Q3								
MM conference								
Yes	745	50.9	2.4	.013	3.0	0.81	0.78	0.85
No	719	49.1	2.7		2.9	0.93	0.88	0.99
Q4								
NCD feedback system								
Yes	672	45.9	2.3	<.001	2.9	0.80	0.76	0.84
No	792	54.1	2.8		3.0	0.92	0.87	0.97
Q5								
Team treatment system								
Yes	1072	73.2	2.5	.011	2.9	0.84	0.81	0.87
No	392	26.8	2.8		3.0	0.92	0.84	1.00
Q6								
Education institutional								
Yes	614	41.9	2.4	<.001	3.0	0.79	0.75	0.83
No	850	58.1	2.8		2.8	0.99	0.93	1.05
Q7								
ICU								
Yes	856	58.5	2.5	.094	3.0	0.82	0.78	0.85
No	608	41.5	2.7		2.6	1.05	0.96	1.13
Q8								
ICT								
Yes	1320	90.2	2.5	.004	3.0	0.84	0.81	0.87
No	144	9.8	3.1		2.8	1.14	0.96	1.32
Q9								
NST								
Yes	1331	90.9	2.5	<.001	3.0	0.84	0.81	0.87
No	133	9.1	3.4		2.9	1.19	0.99	1.38
Q10								
Medical safety committee								
Yes	1410	96.3	2.5	.995	3.0	0.85	0.82	0.88
No	54	3.7	2.5		2.8	0.90	0.68	1.12
Q11								
WHO safe check list								
Yes	1217	83.1	2.5	.010	3.0	0.84	0.81	0.87
No	247	16.9	3.0		2.8	1.05	0.91	1.19
Q12								
BCS‐G								
0	113	7.7	3.3	<.001	3.0	1.10	0.85	1.35
1	351	24.0	3.5		3.1	1.11	1.00	1.22
2≤	1000	68.3	2.4		2.9	0.82	0.79	0.85
Q13								
Surgery volume per year								
<2500	917	62.6	3.0	<.001	2.8	1.08	1.02	1.14
2500‐4999	259	17.7	2.5		2.9	0.86	0.80	0.92
5000‐9999	224	15.3	2.1		3.1	0.70	0.65	0.75
10 000≤	64	4.4	2.3		3.2	0.71	0.62	0.81
Q14								
Certified nurse								
0	149	10.2	3.4	<.001	2.5	1.38	1.08	1.68
1‐5	616	42.1	3.1		3.0	1.04	0.97	1.11
6‐10	373	25.5	2.5		2.9	0.87	0.81	0.92
11≤	326	22.3	2.2		3.0	0.73	0.69	0.77
Q15								
Institutional bed								
<25	6	0.4	8.3	<.001	0.7	12.28	‐4.74	29.31
25‐99	91	6.2	2.4		1.6	1.51	1.03	1.98
100‐499	1032	70.5	2.8		2.9	0.98	0.93	1.02
500≤	335	22.9	2.2		3.0	0.72	0.68	0.76

Abbreviations: BCS‐G, board‐certified surgeon of gastroenterological surgery; CI, confidence interval; ICT, infection control team; ICU, intensive care unit; MM, mortality and morbidity; NCD, National Clinical Database; NST, nutrition support team; O/E, observed/expected; QI, questionnaire item; WHO, World Health Organization.

### 3.1 Differences in profiles of institutional departments across volume groups

The percentages of institutional departments with affirmative responses to the 15 questions across the category of annual case volume are shown in Table 5. The number of institutional departments with <40, 40‐79, 80‐199, 200‐499, and 500 ≤annual cases was 602, 286, 380, 181, and 15, respectively. The frequencies of departments meeting the conditions asked in the questionnaire were low for all items in Group A, in which fewer than half of the departments had a cancer board, held an MM conference, used the NCD feedback system, and held a preoperative conference. In Groups B, C, D, and E, the frequencies of institutional departments without a certified nurse in their institution were 0%‐3.8%, but in Group A, the frequency was high with 22.4%.

**TABLE 5:** Relationship between annual case numbers of the eight major surgical procedures and affirmative response for each QI

Questionnaire item	Annual cases of the eight major surgical procedures									
	<40		40‐79		80‐199		200‐499		500≤	
Number of the department	602		286		380		181		15	
Q1										
Preoperative conference	273	45.3%	193	67.5%	313	82.4%	159	87.8%	15	100.0%
Q2										
Cancer Board	189	31.4%	182	63.6%	342	90.0%	173	95.6%	14	93.3%
Q3										
MM conference	204	33.9%	132	46.2%	243	63.9%	151	83.4%	15	100.0%
Q4										
NCD feedback system	212	35.2%	126	44.1%	208	54.7%	120	66.3%	6	40.0%
Q5										
Team treatment system	371	61.6%	212	74.1%	301	79.2%	173	95.6%	15	100.0%
Q6										
Education institutional	69	11.5%	119	41.6%	254	66.8%	158	87.3%	14	93.3%
Q7										
ICU	171	28.4%	166	58.0%	334	87.9%	170	93.9%	15	100.0%
Q8										
ICT	494	82.1%	264	92.3%	371	97.6%	176	97.2%	15	100.0%
Q9										
NST	497	82.6%	272	95.1%	371	97.6%	176	97.2%	15	100.0%
Q10										
Medical safety committee	565	93.9%	280	97.9%	372	97.9%	178	98.3%	15	100.0%
Q11										
WHO safe check list	402	66.8%	264	92.3%	360	94.7%	176	97.2%	15	100.0%
Q12										
BCS‐G										
0	93	15.4%	14	4.9%	4	1.1%	1	0.6%	1	6.7%
1	249	41.4%	64	22.4%	30	7.9%	8	4.4%	0	0.0%
2≤	260	43.2%	208	72.7%	346	91.1%	172	95.0%	14	93.3%
Q14										
Certified nurse										
0	135	22.4%	11	3.8%	3	0.8%	0	0.0%	0	0.0%
1‐5	394	65.4%	149	52.1%	60	15.8%	13	7.2%	0	0.0%
6‐10	59	9.8%	101	35.3%	177	46.6%	35	19.3%	1	6.7%
11≤	14	2.3%	25	8.7%	140	36.8%	133	73.5%	14	93.3%
Q15										
Institutional bed										
<25	6	1.0%	0	0.0%	0	0.0%	0	0.0%	0	0.0%
25‐99	81	13.5%	7	2.4%	3	0.8%	0	0.0%	0	0.0%
100ー499	511	84.9%	263	92.0%	230	60.5%	26	14.4%	2	13.3%
500≤	4	0.7%	16	5.6%	147	38.7%	155	85.6%	13	86.7%

Abbreviations: BCS‐G, board‐certified surgeon of gastroenterological surgery; ICT, infection control team; ICU, intensive care unit; MM, mortality and morbidity; NCD, National Clinical Database; NST, nutrition support team; QI, questionnaire item; WHO, World Health Organization.

### 3.2 Association of institute volume category with operative outcomes

**FIGURE 1:**
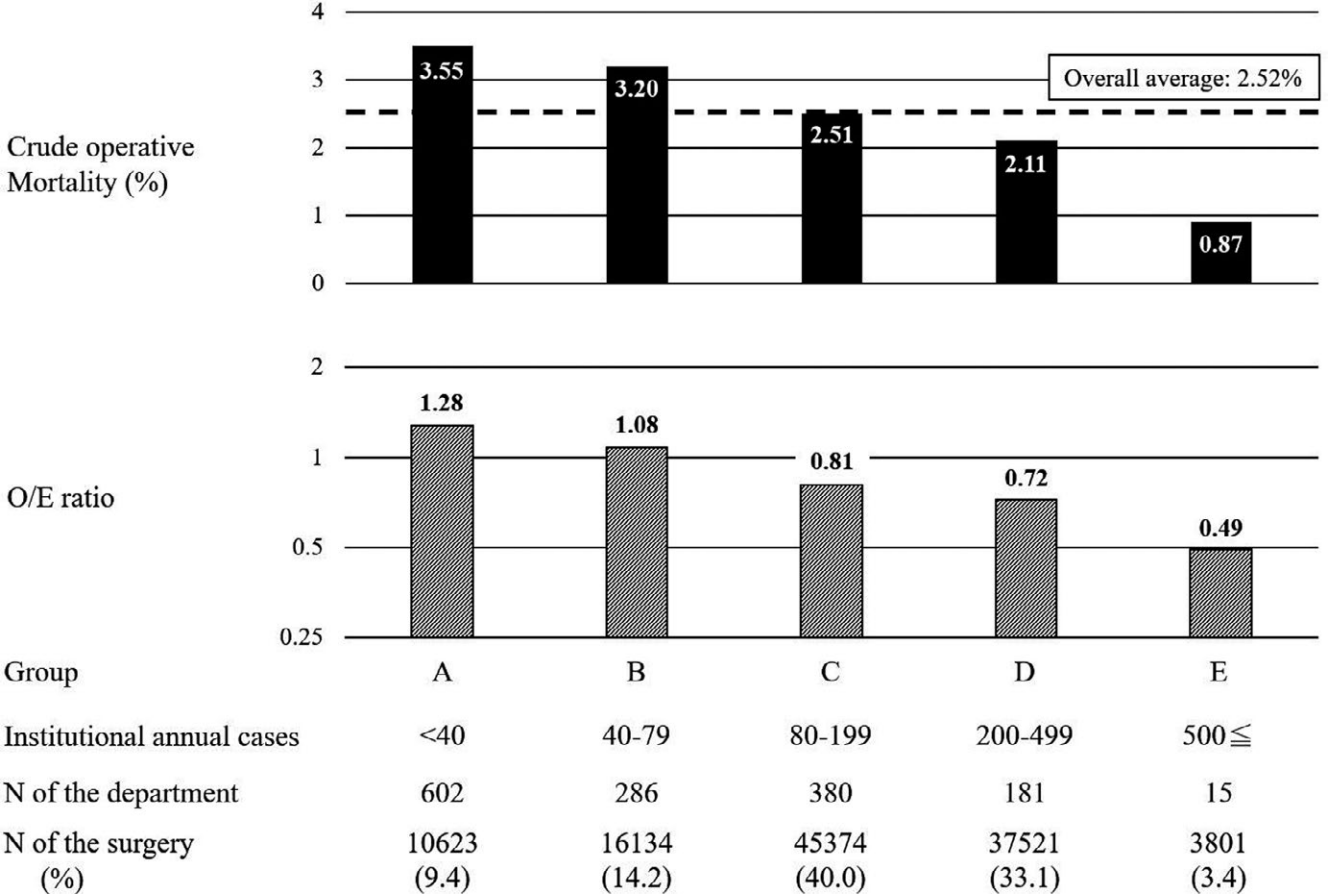
Association of institute category with operative outcomes

Mortality progressively decreased from Group A to Group E (Figure 1). The mortality in Group E was 0.9%, and well below the average mortality of 2.5%. The same tendency was observed for the O/E ratio, in which Groups A and B had ratios of more than 1, and Groups C, D, and E had ratios of less than 1. The O/E ratio in group E was 0.49, which was remarkably low among the five groups.

### 3.3 Improvable conditions and operative outcomes

**FIGURE 2:**
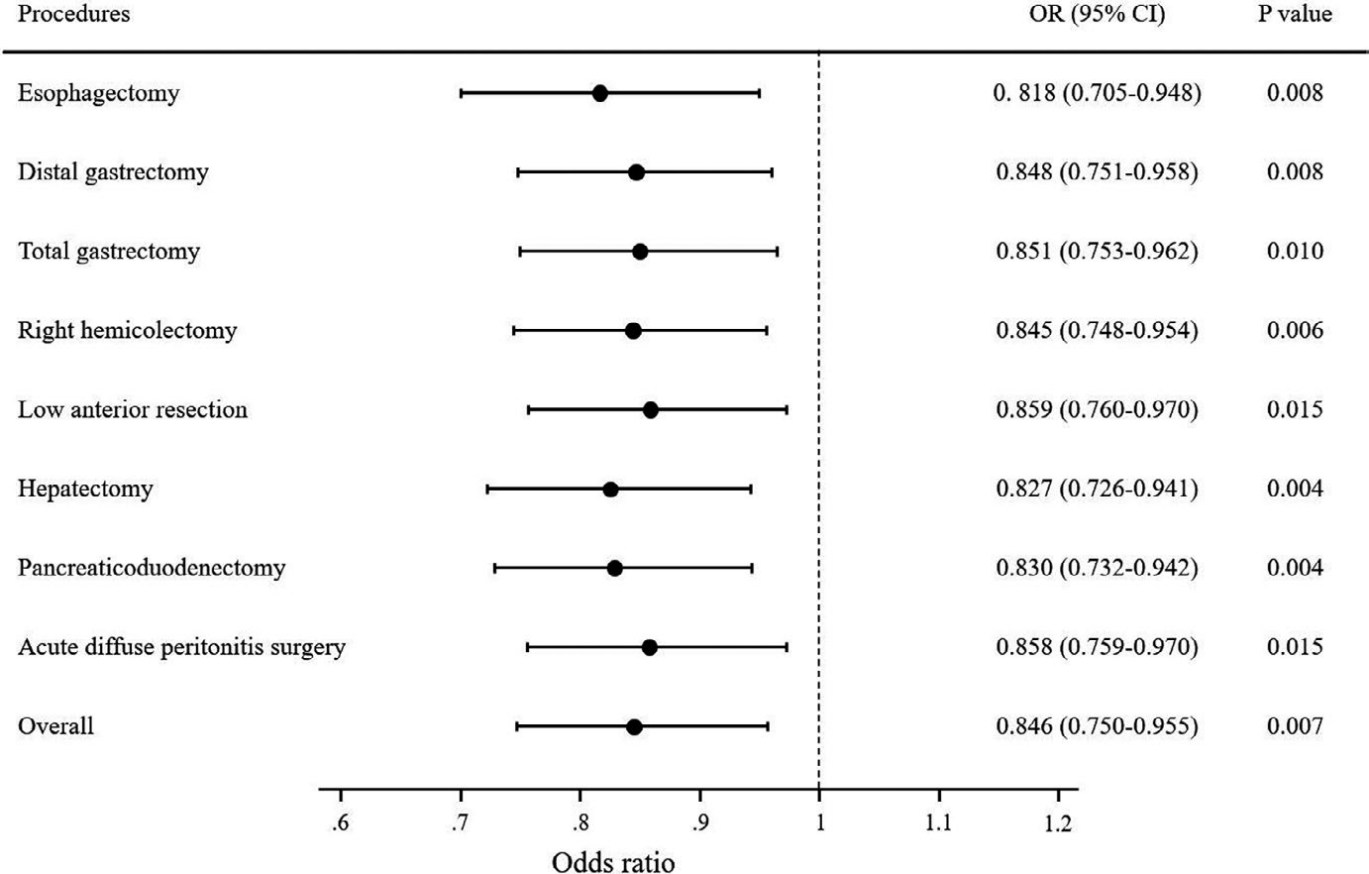
Impact of nine or more conditions met on mortality of eight procedures

Table 6 shows the results of the multivariable logistic regression model for operative mortality with departments categorized based on the number of conditions met. It suggests an increasing trend of mortality odds ratios as departments meeting fewer conditions when compared with departments meeting all of the conditions. It also showed that while the departments meeting nine conditions had mortality odds that are not statistically significantly different from those meeting 10, the departments meeting only eight had statistically significantly higher odds ratio. Based on these findings, we recognized the use of meeting nine conditions among the 10 as being a good indicator for having appropriate structural and process measures for gastroenterological surgery. Figure 2 depicts the association between nine or more conditions met among the 10 improvable conditions and operative mortality. In the overall cohort, meeting nine or more conditions was significantly associated with decreased odds of operative mortality compared with facilities meeting eight or less conditions. When the association was assessed by procedure, the odds of operative mortality were significantly lower for all eight procedures.

**TABLE 6:** Multivariable logistic regression model for operative mortality with facilities categorized based on the number of conditions met

Number of conditions the department met	Number of department	Number of surgery	Observed Mortality (%)	Mortality OR	95%CI		*P* value
					lower	upper	
1 or less	15	106	8.5	6.15	2.72	13.87	<.001
2	39	302	4.0	1.96	0.91	4.23	.09
3	64	953	3.8	2.03	1.40	2.95	<.001
4	78	1717	2.7	1.46	0.98	2.17	.07
5	130	3998	2.6	1.41	1.05	1.88	.02
6	156	5827	3.4	1.61	1.29	2.01	<.001
7	230	13 668	3.0	1.57	1.32	1.88	<.001
8	254	20 110	2.7	1.35	1.14	1.60	<.001
9	280	34 802	2.4	1.17	0.99	1.38	.07
10	218	31 970	2.1	1.00			

Abbreviations: CI, confidence interval; OR, odds ratio.

## 4 DISCUSSION

The JSGS has a well‐organized board system that has been maintained for a long time by board committee members. More than 6000 BCS‐Gs have played central roles in gastroenterological surgery throughout Japan, but it has been difficult to evaluate the quality of BCS‐Gs with regard to operative outcomes because of the small number of cases for which data were previously available. Establishment of the NCD database has enabled us to accurately evaluate the contribution of BCS‐Gs to better operative outcomes. For the first time in our previous study, we demonstrated the positive impact of BCS‐Gs on the outcomes of eight procedures of gastroenterological surgery.^1^

In the context of our present study, we herein investigated the association of the profiles of institutional departments with operative outcomes, using an online questionnaire survey comprising 15 questionnaire items (QIs) on the process for determination of surgical indications or surgical options, institutional department systems for patient safety, and improvement in quality of surgery. We obtained responses from more than half of the institutional departments that received the questionnaire survey.

First, we investigated the correlation of affirmative response or negative response to every QI with operative outcomes, to identify individual items that were significantly positively related to operative outcomes. Thirteen items with affirmative responses among the 15 QIs had a significant positive impact on mortality.

We then focused on the association of institutional volume with operative outcomes, because some studies incorporating risk‐adjusted models and using NCD data have investigated the association between institutional patient volume and operative outcomes for Eso,^22^ DG,^23^ Hx,^24^ and PD.^25^ Prior to the analysis, we classified all institutional departments into five categories based on the JSGS requirements for training institutes. Among them, Group A was defined as institutional departments with fewer than 40 annual cases (120 cases in 3 years) in their institutes. A total of 602 institutional departments belonging to Group A showed two characteristics: poor operative outcomes, demonstrated by high mortality and high O/E ratio; and insufficient institutional department profiles, including poor processes for determining surgical indications or options, lack of sufficient BCS‐Gs and certified nurses, and no use of the NCD feedback system. These results offer evidence that regulation of annual cases by the JSGS is reasonable. However, in Group E, both mortality and O/E ratio were satisfactory, and except for the NCD feedback system, all responses to QIs were affirmative. Furthermore, both the number of BCS‐Gs and number of qualified nurses were satisfactory.

Although it is true that institutional volume has an impact on operative outcomes, we hypothesized that the departments’ structural and procedural characteristics on how they care for their surgical patients are vital for the operative outcomes. On the back of this hypothesis, we selected 10 of the 15 QIs as a first step and focused on the association of number of conditions met with operative outcomes. Ten of 13 QIs having a positive impact on mortality were selected after excluding three QIs related to institutional attributes or facilities. Interestingly, there was a strong positive relationship between number of conditions met and mortality odds ratio. Fifteen institutional departments with the lowest number of conditions met showed the highest mortality and mortality odds ratio. As the number of conditions met increased, operative outcomes improved. In addition, it also showed that while the departments meeting nine conditions had mortality odds that are not statistically significantly different from those meeting 10, the departments meeting only eight had a statistically significantly higher odds ratio.

Multivariable analysis showed that nine or more conditions met among 10 improvable characteristics was a predictor of operative mortality in all eight procedures. It is logical that high‐volume institutes have a high number of conditions met, which was partly shown by the analysis of five categories classified by institutional annual cases. However, using multivariable analysis, we identified an effect of nine or more conditions met as an independent factor after adjustment for the effect of volume, which was significant.

Generally, institutional support systems, such as number of beds, being a teaching institution, and number of ICU beds, as well as various other profile elements established by the efforts by the institutions, including surgical conference, MM conference, cancer board, NST, ICT, and numbers of BCS‐Gs and certified nurses, may contribute to better operative outcomes. However, “certified nurses” in this study included not only nurses for “perioperative nursing” or “critical care,” but also those for “palliative care,” “wound, ostomy and continence nursing,” “cancer chemotherapy nursing,” and others who might not directly influence mortality after surgery. Our throught is that the total number of several types of certified nurses is an important criterion to reflect the policy or culture for better medical care.

We want to place strong importance on clinical conferences, including preoperative, postoperative, and MM conferences, because they can be conducted at any department regardless of the hospital volume. Operative indications and operative methods should be discussed and determined by surgeons and physicians after precise evaluation of patient status, followed by open discussions of various aspects including curability, risks of complications, possible mortality, and age. Furthermore, an ICU and patient safety committee must be present and equipped in institutional departments where surgical treatment is commonly administered. In addition, the NCD feedback system can be leveraged to yield better patient prognosis.

In conclusion, this is the first report to demonstrate a positive association between profiles of institutional departments and operative outcomes. Institutional profiles play a large part in maintaining favorable outcomes of gastrointestinal surgery in Japan, as does the number of BCS‐Gs.

## DISCLOSURE

Funding: This study was partially supported by a research grant from the Ministry of Health, Labour, and Welfare, Japan.

Conflicts of Interest: AT, HK, and HM are affiliated with the Department of Healthcare Quality Assessment at The University of Tokyo. The department is a social collaboration department supported by the National Clinical Database, Johnson & Johnson KK, and Nipro Corporation. The other authors declare no conflicts of interest for this article.

## Supporting information

Appendix S1
