# Supplementary material for: Profiles of institutional departments affect operative outcomes of eight gastroenterological procedures
Source: Ann Gastroenterol Surg. 2021 Feb 20;5(3):304–13. doi: 10.1002/ags3.12431 (PMC8164461; doi:10.1002/ags3.12431)
Supplement: Supplementary file 1 — Appendix S1 [file AGS3-5-304-s001.docx]

**Supplemental Text**

List of patient baseline factors adjusted for in the multivariable analyses for each surgical procedure

Esophagectomy

Sex male, Age category, ADL any assistance, COPD, Weight loss ≥10%, WBC <4500 /μL, Platelet <120,000 /μL, Albumin <3.5 g/dL, BUN <8 mg/dL, Na <138 mEq/L, PT-INR ≥1.25, Cancer metastasis/relapse

Distal gastrectomy

Age category, Emergent surgery, Respiratory distress, Preoperative ADL total assistance, Ascites without control, Previous cerebrovascular disease, Disseminated cancer, Chronic steroid use, Weight loss ≥10%, WBC ≥11,000 /μL, Hematocrit males: <37% females: <32%, Platelets <120,000 /μL, Albumin <3.8 g/dL, Total bilirubin ≥2 mg/dL, AST ≥40 IU/L, ALP ≥340 IU/L, Creatinine ≥1.2 mg/dL, Na <135 mEq/L, PT-INR ≥1.1, APTT ≥40 seconds, ASA ≥grade 3

Total gastrectomy

Age category, Emergent surgery, Respiratory distress, Preoperative ADL any assistance, Ascites, Preoperative dialysis, Cerebrovascular accident, Disseminated cancer, Weight loss ≥10%, WBC <3500 /μL, WBC ≥11,000 /μL, Hematocrit <30%, Albumin <3.0 g/dL, Albumin <3.5 g/dL, Total bilirubin ≥2.0 mg/dL, AST ≥35 IU/L, ALP ≥600 IU/L, Na <138 mEq/L, PT-INR ≥1.25, ASA grade 3, ASA ≥grade 4, Pancreaticosplenectomy

Right hemicolectomy

Age category, Emergent surgery, ADL any assistance, Ascites, Congestive heart failure, Previous PVD surgery, Preoperative dialysis, Cancer multiple metastases, Chronic steroid use, Weight loss ≥10%, Sepsis, WBC ≥9,000 /μL, Hematocrit males: <37% females: <32%, Platelet <80,000 /μL, Platelet <120,000 /μL, Albumin <3 g/dL, Total bilirubin ≥1 mg/dL, AST ≥40 IU/L, BUN <8 mg/dL, BUN ≥60 mg/dL, Na <138 mEq/L, Na ≥145 mEq/L, PT-INR ≥1.1, ASA grade 3, ASA ≥grade 4, Cancer metastasis/relapse

Low anterior resection

Sex male, Age category, BMI ≥30 kg/m^2^, Respiratory distress, preoperative ADL partially assistance, preoperative ADL totally assistance, Ascites, Previous PVD surgery, Disseminated cancer, Preoperative transfusions, Hemoglobin males: <13.5 g/dL females: <12.5 g/dL, Hematocrit males: ≥48% females: ≥42%, Platelet <120,000 /μL, Albumin <2.5 g/dL, AST ≥40 IU/L, Creatinine ≥3 mg/dL, Na <138 mEql/L

Hepatectomy

Sex male, Age category, Emergent surgery, ADL before 30 days any assistance, COPD, Preoperative pneumonia, Ascites, Hemoglobin <10 g/dL, Platelet <80,000 /μL, Platelet <120,000 /μL, Albumin <3.0 g/dL, Albumin <3.5 g/dL , AST ≥35 IU/L, PT-INR ≥1.1, ASA ≥grade 3, Intrahepatic cholangiocarcinoma, Hilar bile duct carcinoma, Gallbladder cancer, Hepatectomy with S1, Hepatectomy with S7, Hepatectomy with S8, Hepatectomy with revascularization, Left trisegmentectomy with S1

Pancreaticoduodenectomy

Age category, BMI ≥25 kg/m^2^, Brinkman index ≥400, Respiratory distress, ADL within 30 days before surgery any assistance, Angina, Weight loss ≥10%, WBC ≥11,000 /μL, Platelet <120,000 /μL, Creatinine ≥3.0 mg/dL, PT-INR ≥1.1, APTT ≥40 seconds, ASA ≥grade 3

Acute diffuse peritonitis surgery

Age category, ADL totally dependent within 30 days before surgery, ADL partial/total assistance immediately before surgery, Preoperative pneumonia, Disseminated cancer, Chronic steroid use, Weight loss ≥10 %, Bleeding disorder without therapy, Preoperative blood transfusion, WBC <4,500 /μL, Hemoglobin males: <13.5 g/dL females:<12.5 g/dL, Hematocrit <30%, Platelet <120,000 /μL, Albumin <2.0 g/dL, Albumin <3.0 g/dL, Total bilirubin ≥3.0 mg/dL, AST ≥35 IU/L, ALP ≥600 IU/L, BUN ≥20 mg/dL, Creatinine ≥2.0 mg/dL, Na <130 mEq/L, CRP ≥10.0 mg/dL ASA grade 3, ASA grade 4, ASA grade 5, Non tumor-bearing

Abbreviations : ADL, activities of daily living; ALP, alkaline phosphatase; APTT, activated partial thromboplastin time; ASA, American Society of Anesthesiologists; AST, aspartate aminotransferase; BMI, body mass index; BUN, blood urea nitrogen; COPD, chronic obstructive pulmonary disease; CRP, C-reactive protein; PT-INR, prothrombin time-international normalized ratio; PVD, peripheral vascular disease; WBC, white blood cell.
